# Supplementary material for: The Gut Microbiome Responds Progressively to Fat and/or Sugar-Rich Diets and Is Differentially Modified by Dietary Fat and Sugar
Source: Nutrients. 2023 Apr 27;15(9):2097. doi: 10.3390/nu15092097 (PMC10180990; doi:10.3390/nu15092097)
Supplement: Supplementary file 1 [file nutrients-15-02097-s001.zip › nutrients-2301401-supplementary.pdf]

Article

The gut microbiome responds progressively to fat and/or sugar-rich diets and is differentially modified by dietary fat and sugar

João Pessoa[1,#], Getachew D. Belew[1,#], Cristina Barroso[1,2], Conceição Egas[1,2,*] and John G. Jones[1]

1 CNC – Center for Neuroscience and Cell Biology, CIBB – Center for Innovative Biomedicine and Biotechnology, University of Coimbra, Coimbra, Portugal
2 Biocant – Technology Transfer Association, Biocant Park, Cantanhede, Portugal
# These authors contributed equally to this work.
* Correspondence: cegas@biocant.pt

Supplementary Materials

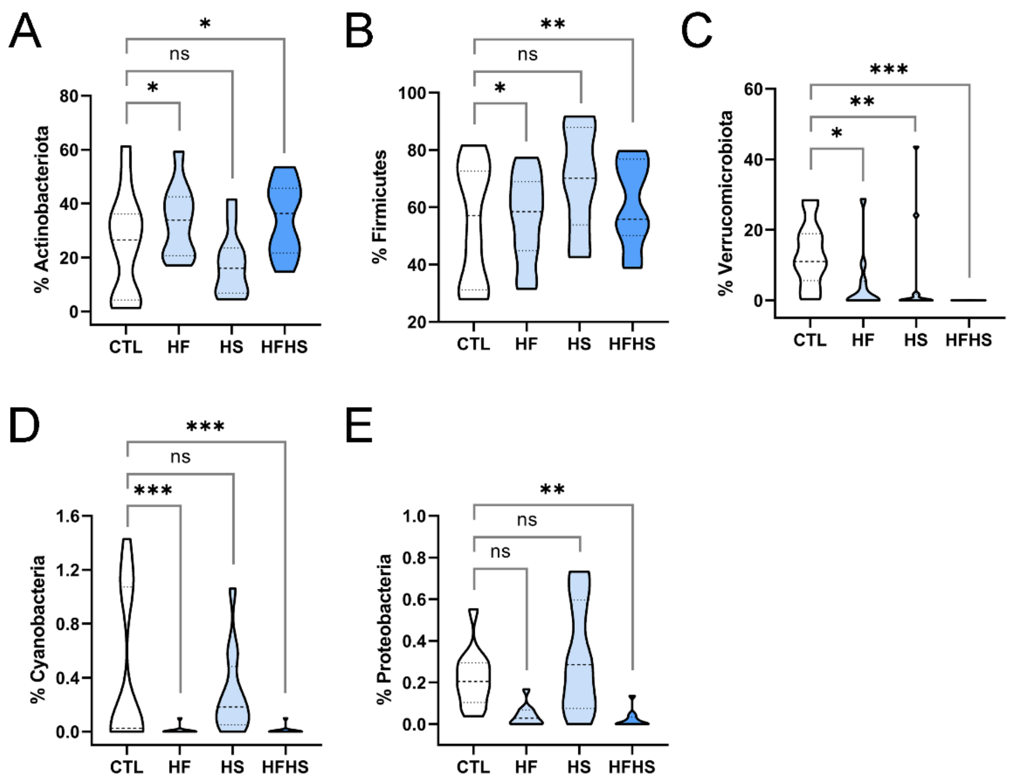

Supplementary figure S1. Bacterial phyla whose percentages in the gut microbiome were affected by high-fat, high-sugar, or high-fat high-sugar diets, at 9 weeks of diet. In each panel, the range of percentages of specific bacterial phyla under control (CTL), high-fat (HF), high-sugar (HS), and high-fat, high-sugar (HFHS) are represented. Dashed and dotted lines represent the median and interquartile separations, respectively. Asterisks indicate statistically significant differences and represent p-values adjusted using the Holm–Bonferroni method (*: 0.05 > p > 0.005; **: 0.005 > p > 0.0005; ***: p < 0.0005; ns: non-significant).

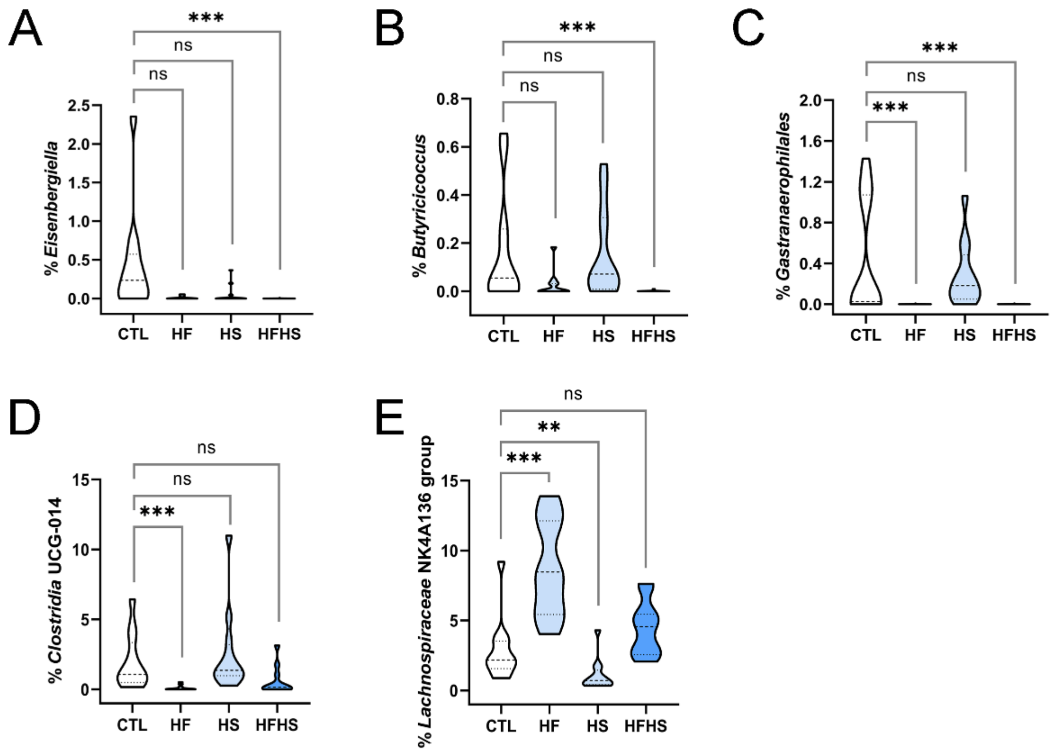

Supplementary figure S2. Bacterial genera whose percentages in the gut microbiome were unstable under the control diet and were further affected by high-fat, high-sugar, or high-fat high-sugar diets. Here, the effects are shown at 9 weeks (A-D) and 18 weeks (E) of diet. In each panel, the range of percentages of a specific bacterial genus under control (CTL), high-fat (HF), high-sugar (HS), and high-fat, high-sugar (HFHS) are represented. Dashed and dotted lines represent the median and interquartile separations, respectively. Asterisks indicate statistically significant differences and represent p-values adjusted using the Holm–Bonferroni method (**: $0.005 > p > 0.0005$; ***: $p < 0.0005$; ns: non-significant).

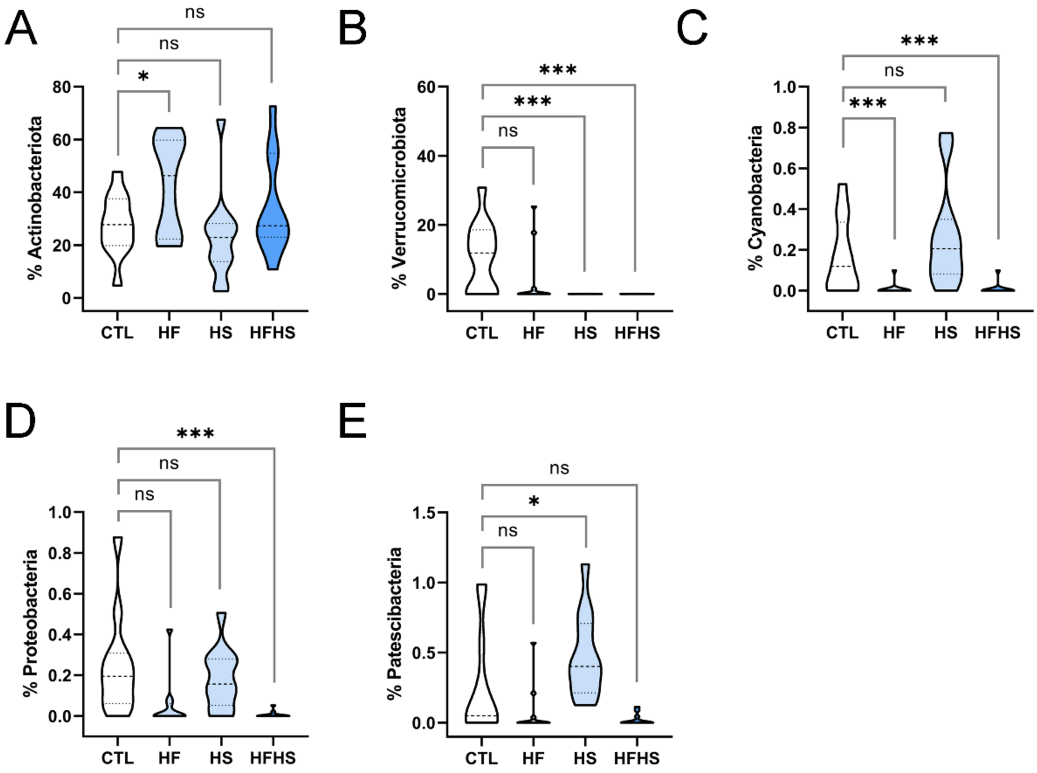

Supplementary figure S3. Bacterial phyla whose percentages in the gut microbiome were affected under high-fat, high-sugar, and high-fat high-sugar diets, at 18 weeks of diet. In each panel, the range of percentages of specific bacterial phyla under control (CTL), high-fat (HF), high-sugar (HS), and high-fat, high-sugar (HFHS) are represented. Dashed and dotted lines represent the median and interquartile separations, respectively. Asterisks indicate statistically significant differences and represent p-values adjusted using the Holm–Bonferroni method (*: 0.05 > p > 0.005; ***: p < 0.0005; ns: non-significant).

Supplementary Table S1. Proximate composition and principal lipid and carbohydrate components of the control and high-fat diets.

Parameters	Components	CTL	HF
	Protein (% by weight)	19	25
**Proximate composition**	Lipids (% by weight)	4	30
	Carbohydrate (% by weight)	71	38
	Fiber (% by weight)	2	3
	Ash (% by weight)	4	4
	Starch (% of total carbohydrate)	92	92
**Specific components**	Sucrose (% of total carbohydrate)	8	8
	Polyunsaturated (% of total lipids)	59	59
	Monounsaturated (% of total lipids)	24	24
	Saturated (% of total lipids)	17	17

Abbreviations used: CTL, control diet; HF, high-fat diet. Diets were purchased from Mucedola s.r.l. (Milan, Italy).

Supplementary table S2. Pairwise comparisons of Chao1 index between specific diet and time conditions calculated with the Mann-Whitney test (related to figure 1E).

	**CTL_0W**	**CTL_9W**	**CTL_18W**	**HF_0W**	**HF_9W**	**HF_18W**	**HFHS_0W**	**HFHS_9W**	**HS_18W**	**HS_0W**	**HS_9W**
**CTL_9W**	0.00952*	-	-	-	-	-	-	-	-	-	-
**CTL_18W**	1	1	-	-	-	-	-	-	-	-	-
**HF_0W**	1	0.00039***	0.1442	-	-	-	-	-	-	-	-
**HF_9W**	1	1	1	0.27076	-	-	-	-	-	-	-
**HF_18W**	0.05237	1	1	0.00918*	1	-	-	-	-	-	-
**HFHS_0W**	1	0.00654*	0.85053	1	0.68523	0.04201*	-	-	-	-	-
**HFHS_9W**	1	1	1	0.00842*	1	1	0.84728	-	-	-	-
**HS_18W**	0.04862*	1	1	0.00225**	1	1	0.03649*	1	-	-	-
**HS_0W**	1	0.0002***	0.44957	1	1	0.00611*	1	0.01357*	0.00185**	-	-
**HS_9W**	1	0.19233	1	0.08542	1	0.22797	1	1	1	0.19233	-
**HS_18W**	0.19233	1	1	0.00293**	1	1	0.06473	1	1	0.00327**	1

Abbreviations used: CTL, control group; HF, high-fat diet; HS, high-sugar diet; HFHS, high-fat, high-sugar diet; 0 weeks, 0W; 9 weeks, 9W; 18 weeks, 18W. Asterisks indicate statistically significant differences and represent p – values adjusted with the Bonferroni method (*: $0.05 > p > 0.005$; **: $0.005 > p > 0.0005$; ***: $p < 0.0005$).

Supplementary table S3. Pairwise comparisons of the Fisher index between specific diet and time conditions calculated with the Mann-Whitney test (related to figure 1F).

	CTL_0W	CTL_9W	CTL_18W	HF_0W	HF_9W	HF_18W	HFHS_0W	HFHS_9W	HS_18W	HS_0W	HS_9W
**CTL_9W**	0.01328*	-	-	-	-	-	-	-	-	-	-
**CTL_18W**	1	1	-	-	-	-	-	-	-	-	-
**HF_0W**	1	0.00039***	0.08542	-	-	-	-	-	-	-	-
**HF_9W**	1	1	1	0.05052	-	-	-	-	-	-	-
**HF_18W**	0.00439**	1	1	0.00037***	1	-	-	-	-	-	-
**HFHS_0W**	1	0.01904*	1	1	0.84728	0.01815*	-	-	-	-	-
**HFHS_9W**	0.37108	1	1	0.00356**	1	1	0.54976	-	-	-	-
**HS_18W**	0.01357*	1	1	0.00131**	1	1	0.05052	1	-	-	-
**HS_0W**	1	0.0002***	0.54735	1	0.23499	0.000098***	1	0.00293**	0.00068**	-	-
**HS_9W**	1	0.15288	1	0.1442	1	0.01357*	1	0.56927	0.23499	0.29786	-
**HS_18W**	0.12077	1	1	0.00185**	1	1	0.1442	1	1	0.00327**	1

Abbreviations used: CTL, control group; HF, high-fat diet; HS, high-sugar diet; HFHS, high-fat, high-sugar diet; 0 weeks, 0W; 9 weeks, 9W; 18 weeks, 18W. Asterisks indicate statistically significant differences and represent p – values adjusted with the Bonferroni method (*: $0.05 > p > 0.005$; **: $0.005 > p > 0.0005$; ***: $p < 0.0005$).

Supplementary table S4. Pairwise PERMANOVA for comparison of time points within each diet calculated with adonis function.

Time pair		p-value
CTL_0W	CTL_9W	0.0014**
CTL_9W	CTL_18W	0.0602
HF_0W	HF _9W	0.0014**
HF _9W	HF _18W	0.1479
HS _0W	HS _9W	0.0014**
HS _9W	HS _18W	0.0196*
HFHS_0W	HFHS _9W	0.0014**
HFHS _9W	HFHS _18W	0.1826

Abbreviations used: CTL, control group; HF, high-fat diet; HS, high-sugar diet; HFHS, high-fat, high-sugar diet; 0 weeks, 0W; 9 weeks, 9W; 18 weeks, 18W. Asterisks indicate statistically significant differences and represent p – values adjusted using the Benjamini-Hochberg procedure (*: 0.05 > p > 0.005; **: 0.005 > p > 0.0005).

Supplementary table S5. Pairwise PERMANOVA for comparison of diets calculated with adonis function.

Diet pair		p-value
CTL	HF	0.0132*
CTL	HFHS	0.003**
CTL	HS	0.004**
HF	HFHS	0.07
HF	HS	0.003**
HFHS	HS	0.0045**

Abbreviations used: CTL, control group; HF, high-fat diet; HS, high-sugar diet; HFHS, high-fat, high-sugar diet. Asterisks indicate statistically significant differences and represent p – values adjusted using the Benjamini-Hochberg procedure (*: 0.05 > p > 0.005; **: 0.005 > p > 0.0005).

Supplementary table S6. Pairwise PERMANOVA for comparison of diets within each time point calculated with adonis function.

Week	Diet pair		p-value
0	CTL_0W	HF_0W	0.243443
0	CTL_0W	HFHS_0W	0.261871
0	CTL_0W	HS_0W	0.449429
0	HF_0W	HS_0W	0.511754
0	HF_0W	HFHS_0W	0.511754
0	HFHS_0W	HS_0W	0.639
9	CTL_9W	HF_9W	0.014943*
9	CTL_9W	HFHS_9W	0.001404**
9	CTL_9W	HS_9W	0.03*
9	HF_9W	HFHS_9W	0.165559
9	HF_9W	HS_9W	0.001404**
9	HFHS_9W	HS_9W	0.001404**
18	CTL_18W	HF_18W	0.001404**
18	CTL_18W	HFHS_18W	0.001404**
18	CTL_18W	HS_18W	0.001404**
18	HF_18W	HFHS_18W	0.003882**
18	HF_18W	HS_18W	0.001404**
18	HFHS_18W	HS_18W	0.011423*

Abbreviations used: CTL, control group; HF, high-fat diet; HS, high-sugar diet; HFHS, high-fat, high-sugar diet; 0 weeks, 0W; 9 weeks, 9W; 18 weeks, 18W. Asterisks indicate statistically significant differences and represent p – values adjusted using the Benjamini-Hochberg procedure (*: 0.05 > p > 0.005; **: 0.005 > p > 0.0005).

Supplementary table S7. Differential abundance of bacterial phyla between 0 and 9 weeks and between 9 and 18 weeks, under the control diet, calculated with ANCOM-BC.

Phylum	0 – 9 weeks	9 – 18 weeks
Actinobacteriota	1	1
Bacteroidota	1	1
Cyanobacteria	0.001327**	1
Deferribacterota	1	1
Desulfobacterota	0***	0***
Firmicutes	0.836339	1
Patescibacteria	1	1
Proteobacteria	1.57E-05***	1
Verrucomicrobiota	0.626909	1
(Unknown)	1	1

Asterisks indicate statistically significant differences and represent p-values adjusted using the Holm–Bonferroni method (**: $0.005 > p > 0.0005$; ***: $p < 0.0005$).

Supplementary table S8. Differential abundance of bacterial genera between 0 and 9 weeks and between 9 and 18 weeks, under the control diet, calculated with ANCOM-BC.

Phylum	Genus	0 – 9 weeks	9 – 18 weeks
Actinobacteriota	*Bifidobacterium*	1	0.046916*
Actinobacteriota	*Coriobacteriaceae* UCG-002	1	1
Actinobacteriota	*Olsenella*	1	1
Actinobacteriota	*Enterorhabdus*	1	1
Actinobacteriota	*Parvibacter*	0.459963	1
Bacteroidota	*Bacteroides*	0***	1
Bacteroidota	*Odoribacter*	0***	0***
Bacteroidota	*Muribaculaceae*	0.459963	1
Bacteroidota	*Prevotellaceae* UCG-001	0***	1
Bacteroidota	*Alistipes*	1	1
Bacteroidota	*Rikenellaceae* RC9 gut group	0***	0.698725
Cyanobacteria	*Gastranaerophilales*	0.019896*	1
Deferribacterota	*Mucispirillum*	1	1
Firmicutes	*Anaeroplasma*	0***	1
Firmicutes	*Faecalibaculum*	0.180051	1
Firmicutes	*Turicibacter*	1	1
Firmicutes	*Lactobacillus*	1	1
Firmicutes	*Christensenellaceae* R-7 group	0***	1
Firmicutes	*Clostridia* UCG-014	0.02095*	1
Firmicutes	*Clostridia* vadinBB60 group	1	1
Firmicutes	Candidatus *Arthromitus*	0.197368	1
Firmicutes	*Clostridium* sensu stricto 1	1	1
Firmicutes	*Anaerofustis*	0***	0***
Firmicutes	*Defluviitaleaceae* UCG-011	0***	0***
Firmicutes	[Eubacterium] fissicatena group	1	0.174052
Firmicutes	[Eubacterium] xylanophilum group	1	1
Firmicutes	*Lachnospiraceae A2*	1	1
Firmicutes	*Acetatifactor*	0.027522*	1
Firmicutes	ASF356	0.632048	1
Firmicutes	*Blautia*	0.002945**	0.341273
Firmicutes	*Eisenbergiella*	0***	1
Firmicutes	GCA-900066575	1	1
Firmicutes	*Lachnoclostridium*	0.000356***	1
Firmicutes	*Lachnospiraceae* FCS020 group	1	1
Firmicutes	*Lachnospiraceae* NK4A136 group	1	0.011254*
Firmicutes	*Lachnospiraceae* UCG-001	0***	1
Firmicutes	*Lachnospiraceae* UCG-006	0.157921	0.438525
Firmicutes	*Marvinbryantia*	1	0.300551
Firmicutes	*Roseburia*	1	1
Firmicutes	*Tuzzerella*	1	1
Firmicutes	*Tyzzerella*	0***	1

Firmicutes	*Monoglobus*	0***	1
Firmicutes	[Eubacterium] coprostanoligenes group	1	0.913246
Firmicutes	*Butyricicoccus*	0.005745*	1
Firmicutes	UCG-009	1	1
Firmicutes	*Colidextribacter*	1	0.810594
Firmicutes	*Intestinimonas*	1	0.321201
Firmicutes	NK4A214_group	1	1
Firmicutes	*Oscillibacter*	1	1
Firmicutes	UCG-005	0***	1
Firmicutes	[Eubacterium] siraeum group	0***	1
Firmicutes	*Anaerotruncus*	0.389384	1
Firmicutes	*Harryflintia*	0.685968	1
Firmicutes	*Ruminococcaceae* Incertae sedis	1	0.199761
Firmicutes	*Paludicola*	1	1
Firmicutes	*Ruminococcaceae*	0***	1
Firmicutes	*Ruminococcus*	0***	1
Firmicutes	UBA1819	1	1
Firmicutes	UCG-010	1	0.378722
Firmicutes	[Eubacterium] brachy group	1	1
Firmicutes	[Eubacterium] nodatum group	0***	0***
Firmicutes	*Anaerovorax*	0.746359	0***
Firmicutes	Family_XIII_AD3011 group	0***	0***
Firmicutes	Family_XIII_UCG-001	0.073102	1
Firmicutes	*Romboutsia*	0***	1
Patescibacteria	Candidatus Saccharimonas	1	1
Proteobacteria	*Parasutterella*	1	1
Verrucomicrobiota	*Akkermansia*	1	1
(Unknown)	(Unknown)	1	1

Asterisks indicate statistically significant differences and represent p-values adjusted using the Holm–Bonferroni method (*: $0.05 > p > 0.005$; **: $0.005 > p > 0.0005$; ***: $p < 0.0005$).
